# Comparative effectiveness of second-line biological therapies for ulcerative colitis and Crohn’s disease in patients with prior failure of anti-tumour necrosis factor treatment

**DOI:** 10.1186/s12876-022-02225-w

**Published:** 2022-03-27

**Authors:** Hye Kyung Hyun, Hyun-Soo Zhang, Jongwook Yu, Eun Ae Kang, Jihye Park, Soo Jung Park, Jae Jun Park, Tae Il Kim, Won Ho Kim, Jae Hee Cheon

**Affiliations:** 1 Department of Internal Medicine, Yongin Severance Hospital, Yonsei University College of Medicine, Seoul, Republic of Korea; 2 Department of Internal Medicine, Severance Hospital, Yonsei University College of Medicine, 50 Yonsei-ro, Seodaemun-gu, Seoul, 120-752 Republic of Korea; 3 Department of Biostatistics, Graduate School of Public Health, Yonsei University, Seoul, Republic of Korea; 4 Department of Biomedical Informatics, Yonsei University College of Medicine, Seoul, Republic of Korea; 5 Department of Internal Medicine and Institute of Gastroenterology, Yonsei University College of Medicine, 50-1 Yonsei-ro, Seodaemun-gu, Seoul, 03722 Republic of Korea

**Keywords:** Ulcerative colitis, Crohn’s disease, Anti-TNF therapy, Vedolizumab, Ustekinumab, Tofacitinib

## Abstract

**Background:**

Therapeutic options for inflammatory bowel disease (IBD) have increased since the introduction of tumour necrosis factor (TNF) inhibitors a few decades ago. However, direct comparisons of the effectiveness of second-line biological agents in patients with ulcerative colitis (UC) and Crohn’s disease (CD) are lacking.

**Methods:**

Patients with UC or CD who experienced anti-TNF treatment failure and subsequently used vedolizumab, ustekinumab, or tofacitinib as a second-line drug were retrospectively recruited. The primary outcomes were the clinical remission rate at week 16 and the cumulative relapse rate 48 weeks after receiving induction therapy.

**Results:**

A total of 94 patients with UC or CD experienced anti-TNF treatment failure and received vedolizumab (UC: 37; CD: 28), ustekinumab (CD: 16), or tofacitinib (UC: 13). The clinical remission rates were not significantly different between the vedolizumab and tofacitinib groups in UC patients (56.8% vs. 46.2%, *p* = 0.509). In CD patients, the clinical remission rates were not significantly different between the vedolizumab and ustekinumab groups (53.6% vs. 50.0%, *p* = 0.820). Moreover, the cumulative rates of clinical relapse were not significantly different between the vedolizumab and tofacitinib groups in UC patients and between the vedolizumab and ustekinumab groups in CD patients (*p* = 0.396 and *p* = 0.692, respectively). Safety profiles were also similar among the treatment groups in both UC and CD patients.

**Conclusions:**

After prior anti-TNF therapy failure, vedolizumab and tofacitinib in UC patients and vedolizumab and ustekinumab in CD patients were not significantly different in terms of the efficacy in inducing and maintaining a clinical response.

**Supplementary Information:**

The online version contains supplementary material available at 10.1186/s12876-022-02225-w.

## Introduction

Inflammatory bowel disease (IBD), including ulcerative colitis (UC) and Crohn’s disease (CD), is caused by abnormal and persistent host immune responses to the gut microbiota or dietary antigens [1]. In the past, the only aim of IBD treatment was to achieve and maintain clinical remission and a clinical response. However, recently, "treat-to-target therapy” has been used to reduce complications and improve patients’ quality of life based not only on clinical symptoms but also on the normalisation of bowel structure and function through mucosal healing [2]. To achieve these therapeutic goals, new biological agents have been introduced, altering the paradigm of therapeutic strategies in IBD patients. During the last 20 years, the introduction of monoclonal antibodies has represented the first revolution in IBD treatment. Tumour necrosis factor (TNF), a proinflammatory cytokine, was found to be one of the key cytokines that initiate and perpetuate intestinal inflammation in IBD, and anti-TNF agents have been considered first-line biological agents for treating moderate to severe UC or CD.

Currently, three types of anti-TNF agents (infliximab, adalimumab, and golimumab) are clinically available, and all of them bind to soluble TNF and membrane-attached TNF in immune cells [3]. Infliximab was the first biological product approved by the Food and Drug Administration in 1998 and the European Medicines Agency in 1999 for the treatment of moderate to severe IBD, and other biosimilar agents are now available [4]. These monoclonal antibodies have proven to be highly effective in the clinical management of CD and/or UC by blocking and neutralising TNF activity.

Despite the high efficacy of anti-TNF agents, approximately 10–40% of patients do not experience an improvement in clinical signs or symptoms after the induction phase, defined as a primary non-response [5]. In addition, a secondary loss of response, defined as worsening of symptoms, can occur as a result of active IBD during maintenance therapy in patients with prior disease control after induction therapy. This can lead to treatment intensification or drug discontinuation in up to 20–50% of patients after 12 months of treatment [6]. Furthermore, anti-TNF agents are associated with an increased risk of infections, such as pneumonia and tuberculosis, and are sometimes associated with malignancies such as hepatosplenic T-cell lymphoma and melanoma [7, 8].

Second-line drugs with different mechanisms have been developed to replace first-line drugs that need to be discontinued or changed due to loss of response or occurrence of side effects. Vedolizumab, a humanised immunoglobulin G1 monoclonal antibody, is the first gut-selective biologic agent to be reported. It inhibits integrin α4β7, a cell-surface glycoprotein variably expressed on circulating B and T lymphocytes, thus inhibiting lymphocyte trafficking from the blood vessels to the intestines [9]. Ustekinumab is a fully human monoclonal antibody targeting the common p40 subunit of interleukin (IL)-12 and IL-23 [10]. Tofacitinib, an oral medication, is a selective small-molecule Janus kinase (JAK) inhibitor that preferentially inhibits JAK 1 and JAK 3 [11].

In previous studies, propensity score-matched analyses or network meta-analyses have been used to compare the efficacy of these second-line drugs [12, 13]; however, there is insufficient real-world data. To date, the VARSITY study, which compared vedolizumab and adalimumab in patients with moderate-severe UC, is the only study on this topic designed as a randomised controlled trial using head-to-head comparison [14]. In addition, there are an insufficient number of studies on actual effectiveness and the predictors of response with respect to second-line drugs. Moreover, there are no data from Asian countries, and differences in anti-TNF responsiveness have been reported among ethnic groups [15].

Accordingly, the aim of this study was to assess comparative effectiveness in terms of the induction of clinical remission and maintenance of clinical response with respect to the following agents: vedolizumab versus tofacitinib in UC patients and vedolizumab versus ustekinumab in CD patients who showed failure of anti-TNF therapy. In addition, we attempted to identify predictors for the induction and maintenance of remission.

## Methods

### Study design and patients

This was a single-centre retrospective study conducted between November 2005 and December 2020. Patients diagnosed with CD or UC aged > 18 years and managed at Severance Hospital, Yonsei University College of Medicine, Seoul, Republic of Korea, were eligible [16, 17]. Patients had moderately to severely active disease, defined by a Mayo score of 6–12 for UC and a Crohn’s disease activity index (CDAI) of 220 or higher for CD. They had also received at least one injection of infliximab, adalimumab, or golimumab as induction treatment, and anti-TNF therapy had been discontinued as the first-line treatment due to a primary non-response, secondary loss of response, occurrence of side effects or malignant tumours, or pregnancy. Those who had not previously used an anti-TNF agent or had been followed-up for < 16 weeks were excluded.

As second-line therapy, patients received intravenous (IV) vedolizumab treatment with an induction regimen of 300 mg at weeks 0, 2, and 6, followed by infusions every 8 weeks for maintenance. In the case of an inadequate response, the interval between the doses of vedolizumab could be reduced to 4 weeks at the discretion of the treating physician. Initial ustekinumab treatment consisted of an IV infusion according to the patient’s body weight (< 55 kg, 260 mg; 55 kg–85 kg, 390 mg; > 85 kg, 520 mg). At week 8, 90 mg of subcutaneous (SC) ustekinumab was administered, followed by a subsequent maintenance SC dose of 90 mg every 8 or 12 weeks, at the discretion of the treating physician. Tofacitinib was administered orally with an induction regimen of 10 mg twice daily for the first 8 weeks. After at least 8 weeks, the treating physician decided whether to maintain the dose at 10 mg or reduce it to 5 mg twice daily, depending on the treatment response. If there was no adequate therapeutic benefit after 16 weeks, treatment was discontinued.

### Outcomes and definitions

The primary outcome of induction therapy was clinical remission at week 16, defined as a Mayo score ≤ 2 for UC and a CDAI < 150 for CD. In the case of non-remission, the response was defined as a Mayo score that had decreased by at least 3 points but that did not correspond to remission, and non-response was defined as a Mayo score that was the same or that had increased [18]. The primary outcome of maintenance therapy was clinical remission or response at 48 weeks after induction therapy. During maintenance therapy, the definition of clinical remission was the same as that for induction therapy. The follow-up duration was calculated from the date of administration of the first induction therapy with vedolizumab or ustekinumab or the initial dose of tofacitinib until the last visit considered in the analysis. Patients who discontinued treatment or had an insufficient response were considered to have treatment failure and were classified as non-responders when determining effectiveness. To determine the predictors of clinical remission, as a secondary outcome, the following variables were investigated: age, sex, body mass index, diagnosis duration, smoking status, intestinal resection history, disease location and behaviour, previous use of anti-TNF agents, disease activity, laboratory variables, and concomitant medications. Patients’ medical records were reviewed, and data were collected to investigate the occurrence of adverse events as another secondary outcome. Adverse events were defined as new diseases or symptoms that occurred after the use of second-line drugs that were not directly related to UC or CD. They were considered serious if they induced prolonged hospitalisation or were life-threatening.

### Statistical analysis

Descriptive analyses were performed to summarise differences in demographic and baseline characteristics between the groups. Continuous variables were compared using the Student’s t-test, and categorical variables were compared using chi-square tests. For the primary outcome of induction therapy, the proportions of patients in clinical remission were compared using logistic regression analyses at week 16. The odds ratio (OR) and 95% confidence interval (CI) were also calculated. Clinical relapse-free survival at 48 weeks after the initiation the second-line drugs was calculated using the Kaplan–Meier method. Univariate analysis was performed to identify any potential factors predictive of remission using log-rank tests. Subsequently, multivariate logistic regression analyses were performed to identify the factors predictive of clinical relapse of CD or UC using Cox regression models. The results are expressed as hazard ratios (HRs) with 95% CIs. Analyses were based on the intention-to-treat principle. Descriptive statistics were used to compare the incidence of adverse events among patients who received vedolizumab, ustekinumab, and tofacitinib. Statistical analyses were performed using IBM SPSS Statistics for Windows, version 25.0 (IBM Corp., Armonk, NY). Statistical significance was defined as a *p* value < 0.05.

## Results

### Baseline patient characteristics

**Fig. 1:**
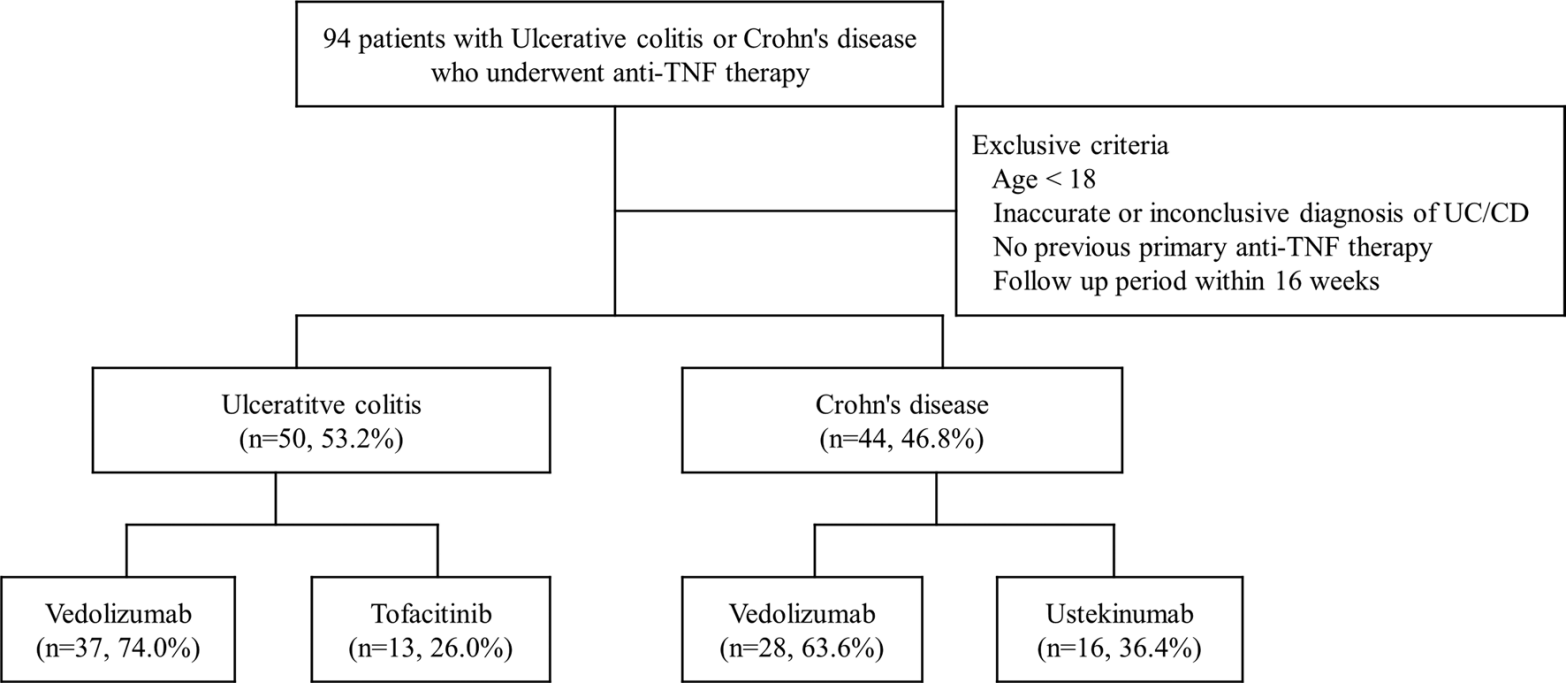
Flowchart of the selection process of the study population. In total, 94 patients were eligible for the study. Patients who met the exclusion criteria were not included, and only patients with previous failure of anti-TNF therapy were enrolled. The study population was stratified into groups according to treatment: vedolizumab or tofacitinib for UC patients and vedolizumab or ustekinumab for CD patients. UC, ulcerative colitis; CD, Crohn’s disease

In total, 94 patients who met the inclusion criteria of our study were enrolled and analysed (Fig. 1). Of these, 50 patients (53.2%) had UC and 44 (46.8%) had CD. The baseline characteristics of the study population are summarised in Tables 1 and 2. Of the 50 patients with UC, 37 (74.0%) received vedolizumab and 13 (26.0%) received tofacitinib after anti-TNF therapy failure. Most of the variables, including clinical and biochemical parameters related to disease activity, did not differ significantly between the groups. The mean patient age was 49.3 years in the entire population; the mean age was significantly higher in the vedolizumab group than in the tofacitinib group (*p* = 0.002). The disease duration of UC was also longer in the vedolizumab group than in the tofacitinib group (41.2 vs. 30.1 years, *p* = 0.013). In addition, differences were observed in smoking status (*p* = 0.002) and concomitant use of an immunomodulator (*p* = 0.000) between patients treated with vedolizumab and tofacitinib (Table 1).

**Table 1:** Baseline characteristics of the patients with UC

Variables	All (n = 50)	Vedolizumab	Tofacitinib	*p* value
		(n = 37, 74.0%)	(n = 13, 26.0%)	
Demographic variables				
Age, years	45.7 ± 14.1	49.3 ± 13.4	35.6 ± 11.1	0.002
Male gender	31 (62.0)	26 (70.3)	5 (38.5)	0.054
Body mass index	21.5 (19.3–23.5)	22.0 (19.4–23.8)	20.6 (18.0–22.1)	0.271
Duration from UC diagnosis, years	38.3 ± 14.1	41.2 ± 14.4	30.1 ± 9.8	0.013
Smoking status at diagnosis				0.002
Never smoked	31 (62.0)	18 (48.6)	13 (100.0)	
Ex-smoker	16 (32.0)	16 (43.2)	0 (0.0)	
Current smoker	3 (6.0)	3 (8.1)	0 (0.0)	
Previous intestinal resection surgery	2 (4.0)	1 (2.7)	1 (7.7)	0.456
UC disease location				0.884
Proctitis (E1)	3 (6.0)	2 (5.4)	1 (7.7)	
Left sided (E2)	19 (38.0)	15 (40.5)	4 (30.8)	
Pancolitis (E3)	28 (56.0)	20 (54.1)	8 (61.5)	
Prior anti TNF therapy use				0.734
1	36 (72.0)	26 (70.3)	10 (76.9)	
≥ 2	14 (28.0)	11 (29.7)	3 (23.1)	
Disease activity index				0.747
Mayo score 6–10	25 (50.0)	19 (51.4)	6 (46.2)	
Mayo score 11–12	25 (50.0)	18 (48.6)	7 (53.8)	
Laboratory variables				
Hemoglobin, g/dL	10.3 (10.0–11.6)	10.3 (10.0–11.8)	10.3 (10.1–11.1)	0.666
Erythrocyte sedimentation rate, mm/h	13.0 (10.8–38.8)	13.0 (11.0–64.5)	13.0 (10.0–48.5)	0.814
Serum C-reactive protein, mg/dL	0.5 (0.3–1.2)	0.5 (0.3–1.0)	0.5 (0.3–11.0)	0.264
Serum albumin, g/dL	2.9 ± 0.7	2.9 ± 0.7	3.0 ± 0.7	0.643
Causes of discontinuation of anti-TNF				0.467
Primary non-response	8 (16.0)	5 (13.2%)	3 (23.1%)	
Secondary non-response	38 (76.0)	28 (75.7%)	10 (76.9%)	
Adverse event	4 (8.0)	4 (10.8%)	0 (0.0%)	
Concomitant medication				
Steroid	18 (36.0)	13 (35.1)	5 (38.5)	1.000
Immunomodulator	21 (42.0)	21 (56.8)	0 (0.0)	< 0.001
5-aminosalicylic acid	49 (98.0)	36 (97.3)	13 (100.0)	1.000

Variables are expressed as the mean (range), median (interquartile range), or n (%)

UC, ulcerative colitis; TNF, tumour necrosis factor

**Table 2:** Baseline characteristics of the patients with CD

Variables	All (n = 44)	Vedolizumab	Ustekinumab	*p* value
		(n = 28, 63.6%)	(n = 16, 36.4%)	
Demographic variables				
Age, years	36.9 (31.3–46.0)	39.2 (31.9–47.6)	34.4 (26.1–40.8)	0.326
Male gender	22 (50.0)	15 (53.6)	7 (43.8)	0.531
Body mass index	18.6 (17.0–20.2)	18.1 (17.0–19.4)	19.6 (17.4–22.4)	0.220
Duration from CD diagnosis, year	21.7 (17.0–28.7)	22.4 (17.0–28.8)	19.7 (17.1–24.6)	0.861
Smoking status at diagnosis				0.220
Never smoked	36 (81.8)	25 (89.3)	11 (68.7)	
Ex-smoker	5 (11.4)	2 (7.1)	3 (18.8)	
Current smoker	3 (6.8)	1 (3.6)	2 (12.5)	
Previous intestinal resection surgery	33 (75.0)	23 (82.1)	10 (62.5)	0.169
Montreal location				0.821
Ileal (L1)	10 (22.7)	7 (25.0)	3 (18.8)	
Colonic (L2)	0 (0.0)	0 (0.0)	0 (0.0)	
Ileocolonic (L3)	33 (75.0)	20 (71.4)	13 (81.3)	
Isolated upper GI disease (L4)	1 (2.3)	1 (3.6)	0 (0.0)	
Montreal disease behavior				0.007
Nonstricturing, nonpenetrating (B1)	13 (29.5)	4 (14.3)	9 (56.2)	
Stricturing (B2)	12 (27.3)	11 (39.3)	1 (6.3)	
Penetrating (B3)	19 (43.2)	13 (46.4)	6 (37.5)	
Perianal disease modifier (p)	33 (75.0)	21 (75.0)	12 (75.0)	1.000
Prior anti TNF therapy use				0.690
1	23 (52.3)	14 (50.0)	9 (56.3)	
≥ 2	21 (47.7)	14 (50.0)	7 (43.8)	
Crohn's disease activity index				0.013
220 ≤ CDAI < 450	40 (90.9)	28 (100.0)	12 (75.0)	
CDAI ≥ 450	4 (9.1)	0 (0.0)	4 (25.0)	
Endoscopic disease activity				0.711
SES-CD score 3–6	14 (31.8)	10 (38.5)	4 (26.7)	
SES-CD score 7–15	23 (52.3)	14 (53.8)	9 (60.0)	
SES-CD score > 15	4 (9.1)	2 (7.7)	2 (13.3)	
Laboratory variables				
Hemoglobin, g/dL	10.0 (10.0–11.0)	10.0 (10.0–10.5)	10.0 (10.0–12.6)	0.112
Erythrocyte sedimentation rate, mm/h	44.5 (10.0–102.5)	33.0 (10.0–102.3)	68.0 (10.0–102.5)	0.836
Serum C-reactive protein, mg/dL	0.5 (0.3–4.8)	0.4 (0.2–1.1)	0.8 (0.4–10.9)	0.777
Serum albumin, g/dL	2.5 ± 0.8	2.3 ± 0.6	2.9 ± 1.0	0.026
Causes of discontinuation of anti-TNF				0.482
Primary non-response	5 (11.4)	2 (7.1%)	3 (18.8%)	
Secondary non-response	31 (70.5)	21 (75.0%)	10 (62.5%)	
Adverse event	8 (18.1)	5 (17.9%)	3 (18.8%)	
Concomitant medication				
Steroid	27 (61.4)	20 (71.4)	7 (43.8)	0.070
Immunomodulator	30 (68.2)	16 (57.1)	14 (87.5)	0.038

Variables are expressed as the mean (range), median (interquartile range), or n (%)

CD, Crohn’s disease; TNF, tumour necrosis factor; CDAI, Crohn’s disease activity index; SES-CD, Simple endoscopic score for Crohn’s disease

Of the 44 patients with CD, 28 (63.6%) received vedolizumab and 16 (36.4%) received ustekinumab after prior failure of anti-TNF therapy. Baseline characteristics between the groups were similar. According to the Montreal classification, 4 (14.3%) patients had nonstricturing, nonpenetrating type disease (B1), 11 (39.3%) had stricturing type disease (B2), and 13 (46.4%) had penetrating type disease (B3) in the vedolizumab group, while 9 (56.2%) patients had nonstricturing, nonpenetrating type disease (B1), 1 (6.3%) had stricturing type disease (B2), and 6 (37.5%) had penetrating type disease (B3) in the ustekinumab group (*p* = 0.007). Differences between vedolizumab- and ustekinumab-treated patients were observed in terms of CDAI scores that were ≥ 220 but < 450 (100% vs. 75.0%, *p* = 0.013), serum albumin levels (2.3 vs. 2.9 g/dL, *p* = 0.026), and concomitant use of an immunomodulator (57.1% vs. 87.5%, *p* = 0.038) (Table 2).

In our cohort, the major reasons for discontinuing anti-TNF therapy were a primary non-response, secondary loss of response, and occurrence of adverse events. Reasons for discontinuation were not significantly different between the vedolizumab and tofacitinib groups in UC patients (*p* = 0.467) or between the vedolizumab and ustekinumab groups in CD patients (*p* = 0.482) (Tables 1, 2).

### Comparison of clinical remission induction and cumulative relapse rates between secondary agents

**Fig. 2:**
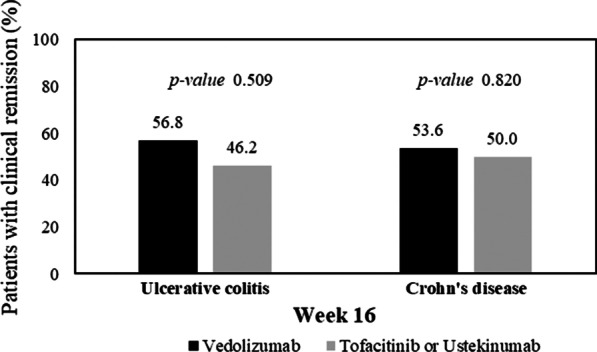
Comparison of induction of clinical remission among second-line drugs. There were no significant differences in clinical remission rates between the vedolizumab and tofacitinib groups in UC patients (*p* = 0.509) or between the vedolizumab and ustekinumab groups in CD patients (*p* = 0.820) at week 16 after the initiation of induction therapy. UC, ulcerative colitis; CD, Crohn’s disease

At week 16, the rate of clinical remission was not significantly different between the vedolizumab (21/37, 56.8%) and tofacitinib (6/13, 46.2%) groups in UC patients (*p* = 0.509) and between the vedolizumab (15/28, 53.6%) and ustekinumab (8/16, 50.0%) groups in CD patients (*p* = 0.820) (Fig. 2).

**Fig. 3:**
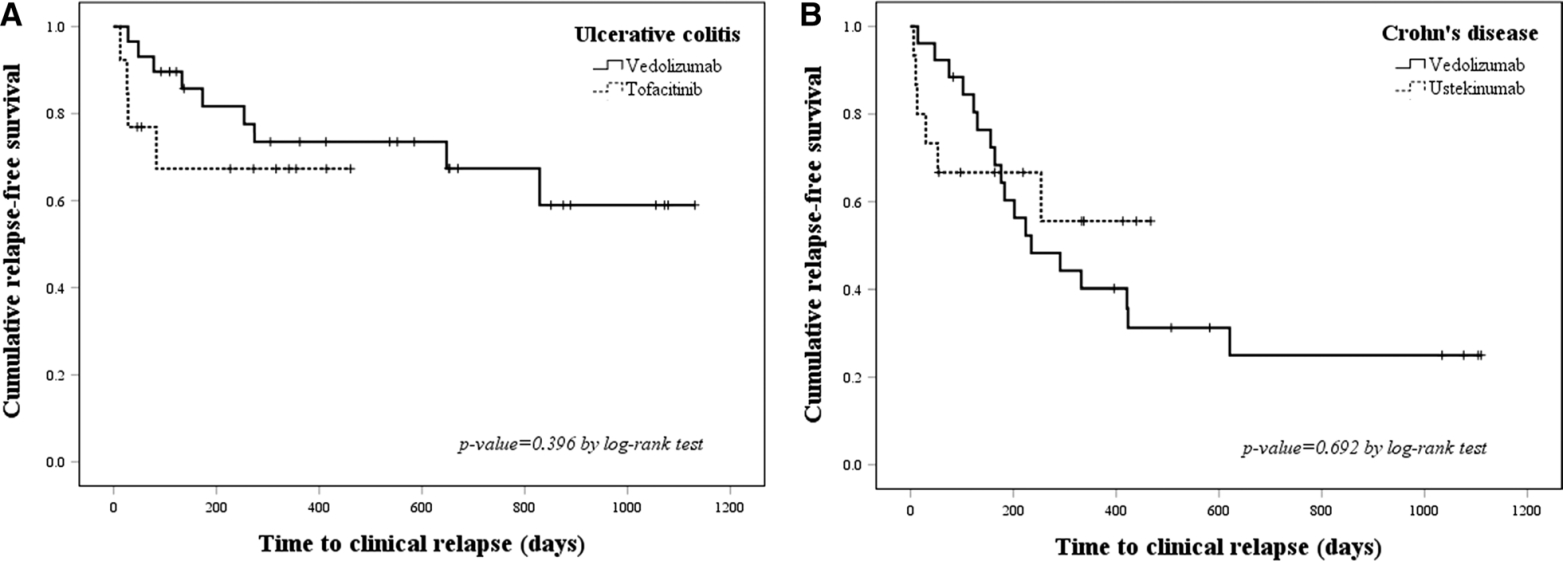
Comparison of cumulative rates of the maintenance of clinical response between **A** vedolizumab and tofacitinib in UC patients and **B** vedolizumab and ustekinumab in CD patients. There were no significant differences in the cumulative rates of the maintenance of clinical response between the vedolizumab and tofacitinib groups in UC patients (*p* = 0.396) or the vedolizumab and ustekinumab groups in CD patients (*p* = 0.692) as of week 48 of maintenance therapy. UC, ulcerative colitis; CD, Crohn’s disease

At 48 weeks after induction, there was no significant difference in the cumulative rate of the maintenance of clinical response between the vedolizumab and tofacitinib groups in UC patients (*p* = 0.396) or between the vedolizumab and ustekinumab groups in CD patients (*p* = 0.692) (Fig. 3).

### Predictors of clinical remission and clinical relapse

At week 16, our multivariate analysis showed that younger age (< 40 years) (OR, 16.620; 95% CI 1.192–231.681), a longer duration from diagnosis (OR, 1.145; 95% CI 1.035–1.267), a Mayo score of 11 to 12 (OR, 7.267; 95% CI 1.352–39.067), and a higher erythrocyte sedimentation rate (ESR) (> 13.0 mm/h) (OR, 1.028; 95% CI 1.001–1.056) independently predicted non-clinical remission in UC patients. In CD patients, multivariate analysis showed that older age (> 40 years) at the time of CD diagnosis (OR, 26.826; 95% CI 1.044–689.453) was significantly predictive of clinical remission. In addition, a longer duration from CD diagnosis (OR, 1.140; 95% CI 1.001–1.29) and concomitant use of a steroid (OR, 22.176; 95% CI 1.800–273.211) independently predicted non-clinical remission (Additional file 1: Tables S1 and S2).

As for clinical relapse, multivariate analysis showed that younger age (< 40 years) (HR, 5.330; 95% CI 1.118–25.415), presence of pancolitis (E3) (HR, 4.896; 95% CI 1.074–22.326), and concomitant use of steroids (HR 3.846; 95% CI 1.044–14.165) independently predicted clinical relapse in UC patients. Moreover, multivariate analysis showed that a higher C-reactive protein (CRP) level (> 0.5 mg/dL) (HR, 1.036; 95% CI 1.016–1.055) and concomitant use of steroids (HR 8.448; 95% CI 2.155–33.113) independently predicted clinical relapse in CD patients (Additional file 1: Tables S3 and S4).

### Safety

None of the patients died from side effects of the study drugs during follow-up. There were 11 (16.9%) adverse events in the vedolizumab group, 3 (18.8%) in the ustekinumab group, and 4 (30.8%) in the tofacitinib group. There were 3 patients with pruritus; 2 with skin rash; 1 with arthralgia; 2 with herpes zoster; and 3 with ear, nose, or throat infections in the vedolizumab group. There were 3 patients each with paraesthesia, a flu-like illness, and urinary tract infection in the ustekinumab group. There were 4 patients each with headache, paraesthesia, arthralgia, and flu-like illness in the tofacitinib group (Table 3).

**Table 3:** Safety of vedolizumab, ustekinumab, and tofacitinib in patients with UC and CD refractory to anti-TNF therapy

Variables	Vedolizumab	Ustekinumab	Tofacitinib
	(n = 65, 69.2%)	(n = 16, 17.0%)	(n = 13, 13.8%)
Headache			1
Paraesthesia		1	1
Pruritus	3		
Rash	2		
Arthralgia	1		1
Herpes zoster	2		
Flu or flu-like illness	1	1	1
Rhinitis	1		
Oral infection	1		
Urinary tract infection		1	
Adverse event total	11 (16.9)	3 (18.8)	4 (30.8)

CD, Crohn's disease; TNF, tumour necrosis factor; UC, ulcerative colitis

## Discussion

Few studies to date have directly compared secondary drugs for IBD treatment. For patients with moderate to severe UC or CD showing failure of first-line anti-TNF therapy, choosing the best option for secondary treatment is critical, based on the efficacy and safety of the available second-line agents. This study focused on comparing the effectiveness of vedolizumab versus tofacitinib in patients with UC and vedolizumab versus ustekinumab in patients with CD who received second-line drugs because they were refractory or intolerant to anti-TNF therapy. The therapeutic goals for IBD patients can be reached by selecting an effective drug for second-line treatment [19].

Overall, the baseline characteristics were similar, but since our study was based on real-world data, there were inevitable differences in baseline characteristics between the groups. In the group using vedolizumab, the disease duration was relatively longer, and patients were older in the UC group. This reflects the known safety of vedolizumab in elderly patients and the fact that elderly people often have a longer disease duration [20]. The concomitant use of tofacitinib and immunomodulators is contraindicated; therefore, only patients who received vedolizumab showed concomitant use of immunomodulators in the baseline analysis of UC patients [21]. Likewise, for patients with CD, most of the baseline characteristics were similar between the two drug groups, with a few exceptions. The CDAI score, for instance, was higher in the ustekinumab group than in the vedolizumab group. In actual clinical practice, vedolizumab is administered more commonly to those with moderate disease activity than to those with severe disease activity since it takes time for vedolizumab to be effective. Analysis of data from observational cohorts, including those from clinical trials, suggests that the median onset of vedolizumab action, which involves the inhibition of leukocyte trafficking to the gut mucosa, may take 10 weeks in UC patients and as much as 14 weeks in CD patients [22, 23]. In addition, the concomitant use of immunomodulators was more often found in patients with severe disease activity. Since these differences in baseline characteristics could affect drug selection, caution must be taken when comparing these drugs. However, even with these considerations, there were no significant differences in clinical remission and relapse rates between the second-line drugs. Therefore, it is necessary to select an appropriate drug as the second-line biological agent for each patient with UC or CD based on not only the effectiveness of the drug itself but also the safety and onset of the action of the drug; it is also important to consider whether an immunomodulator is being used concomitantly.

Several previous studies have compared these drugs. Alric et al. found that for patients with CD refractory or intolerant to anti-TNF agents, the clinical remission (54.4% vs. 38.3%, OR 1.92; 95% CI 1.09–3.39) and treatment persistence rates (71.5% vs. 49.7%, OR 2.54; 95% CI 1.40–4.62) at week 48 were higher in the ustekinumab group than in the vedolizumab group [24]. The Dutch Initiative on Crohn and Colitis (ICC) Registry, a prospective multicentre analysis, reported that tofacitinib was still effective in achieving clinical remission of UC after anti-TNF and/or vedolizumab failure [25]. This supports the finding that more effective clinical remission can be achieved using second-line drugs with mechanisms different that those of anti-TNF agents. However, no significant differences between vedolizumab and tofacitinib in UC patients or between vedolizumab and ustekinumab in CD patients were found with respect to the achievement of clinical remission in patients who showed failure of anti-TNF therapy. These discrepancies may be due to racial differences or differences in treatment indications between countries. In addition, the number of patients enrolled is often small, and differences in study design may yield discrepancies in results. In the future, large-scale prospective studies and registration studies are needed, especially in Asia.

In patients with both UC and CD, at week 16, the drug response rates were lower in those with a longer disease duration [26,27]. Moreover, UC patients with severe disease activity had a lower drug response rate. In the US VICTORY Consortium study, clinical remission (HR 0.54; 95% CI 0.31–0.95) and mucosal healing (HR 0.54; 95% CI 0.31–0.95) were found less commonly in CD patients with severe disease activity than in those with moderate disease activity [26]. In patients with UC, at week 48, younger age and extensive disease were associated with a shorter time to relapse. A prospective longitudinal study in patients with UC reported that younger patients, particularly those in the 20–30 year age group, had a shorter time to relapse [27]. This is consistent with the results of previous studies showing that in patients with extensive UC (E3), pancolitis was associated with a higher cumulative probability of relapse, indicating a poor prognosis [28]. High CRP levels, a marker of inflammation, was correlated with disease activity in CD patients. This is consistent with a well-known finding that high CRP levels increase the clinical relapse rate [29]. A recently emerging ‘top-down’ therapeutic approach for CD includes the early use of immunomodulators [30]. In our study, patients with more severe CD were more commonly taking immunomodulators, and the results show that the prognosis was poor for these patients. In addition, it is well known that steroid use itself is related to high disease activity and poor prognosis [30, 31].

The safety of vedolizumab, ustekinumab, and tofacitinib observed in our study was similar to that previously reported. Sands et al. showed that few differences were found between the adalimumab and vedolizumab treatment groups [14]. In addition, Biemans et al. found that there were no differences in adverse events, infection rates, or hospitalisation rates between the vedolizumab and ustekinumab treatment groups [32]. No serious side effects leading to severe infection or death were reported, and little difference was observed between the trial groups in the most commonly reported side effect measures.

Our study findings have several clinical implications. First, we were able to reliably assess the effectiveness of vedolizumab versus ustekinumab in UC patients and vedolizumab versus tofacitinib in CD patients after anti-TNF therapy failure in a real-life setting. Second, we followed-up the patients for a 48-week period, which was sufficient to reliably assess the clinical response in patients who had previously used anti-TNF agents. Moreover, the design of this study intentionally did not favour one of the two options for second-line therapy. In the future, in addition to clinical studies, translational studies are needed to identify predictive biochemical markers using biological samples and larger cohort studies including biochemical factors and endoscopic findings are warranted.

This study has several limitations. First, the standardised choice of treatment could not be determined due to the retrospective observational design. Second, although we tried to increase the overall sample size of our study, the number of patients receiving second-line drugs after previous anti-TNF therapy failure was inevitably small, which may have biased the results. Third, therapeutic drug monitoring and changes in dosages during maintenance therapy were not systematically evaluated in our study. Fourth, vedolizumab group in CD patients had a more severe phenotype, which might affect clinical outcomes. Finally, no objective or mandatory criteria were used for evaluating drug-related side effects.

## Conclusion

The results of our study, which included patients with moderate to severe UC and CD, showed no differences in effectiveness between tofacitinib and vedolizumab in UC patients or between ustekinumab and vedolizumab in CD patients with prior failure of anti-TNF therapy. Safety outcomes were comparable. In the future, more real-world data and head-to-head trials are needed to further investigate the comparative efficacy of ustekinumab, vedolizumab, and tofacitinib as second-line therapeutic agents after anti-TNF failure.

## Supplementary Information

**Additional file 1: Supplementary Table 1–4.** The predictors of the adjusted cumulative clinical remission rate versus clinical response or non-response were analyzed at 16 and 48 weeks in patients with UC or CD.

## Data Availability

The datasets used and/or analyzed during the current study are available from the corresponding author on reasonable request.
