# Supplementary material for: Comparative effectiveness of second-line biological therapies for ulcerative colitis and Crohn’s disease in patients with prior failure of anti-tumour necrosis factor treatment
Source: BMC Gastroenterol. 2022 Mar 27;22:143. doi: 10.1186/s12876-022-02225-w (PMC8958783; doi:10.1186/s12876-022-02225-w)
Supplement: Supplementary file 1 — Additional file 1: Supplementary Table 1–4. The predictors of the adjusted cumulative clinical remission rate versus clinical response or non-response were analyzed at 16 and 48 weeks in patients with UC or CD. [file 12876_2022_2225_MOESM1_ESM.doc]

| **Supplementary Table 1. Predictors of clinical remission in UC patients at 16 weeks (remission vs. response/non-response)** | | | | | | | |
| --- | --- | --- | --- | --- | --- | --- | --- |
|  | Univariate | | | | Adjusted Response/Non-response | | |
| Variables |  |  |  |  | Multivariate* | | |
|  | OR | 95% CI | | *P* value | OR | 95% CI | *P* value |
| Demographic variables |  | |  |  |  |  |  |
| Age ≥ 40 years |  | | ( Reference ) |  |  | ( Reference ) |  |
| Age < 40 years | 1.308 | | 0.420-4.071 | 0.643 | 16.620 | 1.192-231.681 | 0.037 |
| Male gender | 1.829 | | 0.568-5.882 | 0.311 | - | - | - |
| Body mass index | 0.926 | | 0.761-1.126 | 0.441 | 0.784 | 0.569-1.080 | 0.136 |
| Duration from UC diagnosis, years | 1.031 | | 0.989-1.074 | 0.157 | 1.145 | 1.035-1.267 | 0.009 |
| Smoking status at diagnosis |  | |  |  |  |  |  |
| Never smoked |  | | ( Reference ) |  |  |  |  |
| Ex-smoker | 2.306 | | 0.599-6.922 | 0.255 | - | - | - |
| Current smoker | 3.167 | | 0.258-38.845 | 0.367 | - | - | - |
| Previous intestinal resection surgery | 1.182 | | 0.070-20.014 | 0.908 | - | - | - |
| UC disease location |  | |  |  |  |  |  |
| Proctitis (E1) |  | | ( Reference ) |  |  |  |  |
| Left sided (E2) | 2.222 | | 0.171-28.856 | 0.542 | - | - | - |
| Pancolitis (E3) | 1.500 | | 0.121-18.540 | 0.752 | - | - | - |
| Prior anti TNF therapy use |  | |  |  |  |  |  |
| 1 |  | | ( Reference ) |  |  |  |  |
| ≥2 | 0.358 | | 0.095-1.355 | 0.130 | - | - | - |
| Disease activity index |  | |  |  |  |  |  |
| Mayo score 6-10 |  | | ( Reference ) |  |  | ( Reference ) |  |
| Mayo score 11-12 | 4.571 | | 1.383-15.109 | 0.013 | 7.267 | 1.352-39.067 | 0.021 |
| Laboratory variables |  | |  |  |  |  |  |
| Hemoglobin, g/dL | 1.200 | | 0.764-1.884 | 0.429 | 1.351 | 0.645-2.831 | 0.426 |
| Erythrocyte sedimentation rate, mm/hr | 1.012 | | 0.997-1.027 | 0.115 | 1.028 | 1.001-1.056 | 0.044 |
| Serum C-reactive protein, mg/dL | 1.007 | | 0.986-1.028 | 0.532 | 0.977 | 0.946-1.008 | 0.141 |
| Serum albumin, g/dL | 1.728 | | 0.727-4.107 | 0.215 | 4.026 | 0.748-21.681 | 0.105 |
| Concomitant medication |  | |  |  |  |  |  |
| Steroid | 0.907 | | 0.284-2.893 | 0.869 | - | - | - |
| Immunomodulator | 0.574 | | 0.183-1.801 | 0.342 | - | - | - |
| Vedolizumab versus Tofacitinib | 1.531 | | 0.430-5.451 | 0.511 | 3.263 | 0.51520.665 | 0.209 |
| UC, ulcerative colitis; TNF, tumour necrosis factor; OR, odd ratio; CI, confidence interval *Adjusted for age, body mass index, disease duration, Mayo score, laboratory variables, and the use of vedolizumab versus tofacitinib | | | | | | | |

| **Supplementary Table 2. Predictors of clinical remission in CD patients at 16 weeks (remission vs. response/non-response)** | | | | | | |
| --- | --- | --- | --- | --- | --- | --- |
|  | Univariate | | | Adjusted Response/Non-response | | |
| Variables |  |  |  | Multivariate* | | |
|  | OR | 95% CI | *P* value | OR | 95% CI | *P* value |
| Demographic variables |  |  |  |  |  |  |
| Age ≥ 40 years |  | ( Reference ) |  |  | ( Reference ) |  |
| Age < 40 years | 1.923 | 0.548-6.748 | 0.307 | 26.826 | 1.044-689.453 | 0.047 |
| Male gender | 1.200 | 0.367-3.922 | 0.763 | 5.471 | 0.668-44.799 | 0.113 |
| Body mass index | 1.018 | 0.850-1.219 | 0.847 | - | - | - |
| Duration from CD diagnosis, years | 1.014 | 0.963-1.067 | 0.596 | 1.140 | 1.001-1.299 | 0.047 |
| Smoking status at diagnosis |  |  |  |  |  |  |
| Never smoked |  | ( Reference ) |  |  |  |  |
| Ex-smoker & Current smoker | 0.114 | 0.013-1.027 | 0.053 | - | - | - |
| Previous intestinal resection surgery | 1.129 | 0.287-4.441 | 0.862 | - | - | - |
| Montreal location |  |  |  |  |  |  |
| Ileal (L1), Colonic (L2), Isolated upper GI disease (L4) | | ( Reference ) |  |  | ( Reference ) |  |
| Ileocolonic (L3) | 1.094 | 0.299-4.006 | 0.892 | 4.145 | 0.423-40.635 | 0.222 |
| Montreal disease behavior |  |  |  |  |  |  |
| Nonstricturing, nonpenetrating (B1) |  | ( Reference ) |  |  |  |  |
| Stricturing (B2) | 0.583 | 0.115-2.952 | 0.515 | - | - | - |
| Penetrating (B3) | 1.604 | 0.387-6.641 | 0.514 | - | - | - |
| Perianal disease modifier (p) | 0.694 | 0.176-2.734 | 0.602 | - | - | - |
| Prior anti TNF therapy use |  |  |  |  |  |  |
| 1 |  | ( Reference ) |  |  | ( Reference ) |  |
| ≥2 | 1.430 | 0.436-4.694 | 0.555 | 5.086 | 0.620-41.715 | 0.130 |
| Crohn's disease activity index |  |  |  |  |  |  |
| 220 ≤ CDAI < 450 |  | ( Reference ) |  |  |  |  |
| CDAI ≥ 450 | 3.667 | 0.351-38.345 | 0.278 | - | - | - |
| Endoscopic disease activity |  |  |  |  |  |  |
| SES-CD score 3-6 |  | ( Reference ) |  |  |  |  |
| SES-CD score ≥ 7 | 1.077 | 0.296-3.917 | 0.910 | - | - | - |
| Laboratory variables |  |  |  |  |  |  |
| Hemoglobin, g/dL | 0.838 | 0.520-1.349 | 0.466 | - | - | - |
| Erythrocyte sedimentation rate, mm/hr | 1.011 | 0.998-1.024 | 0.104 | - | - | - |
| Serum C-reactive protein, mg/dL | 1.001 | 0.978-1.024 | 0.966 | - | - | - |
| Serum albumin, g/dL | 0.669 | 0.309-1.445 | 0.306 | - | - | - |
| Concomitant medication |  |  |  |  |  |  |
| Steroid | 3.491 | 0.956-12.749 | 0.059 | 22.176 | 1.800-273.211 | 0.016 |
| Immunomodulator | 1.155 | 0.610-2.187 | 0.659 | 4.039 | 0.485-33.661 | 0.197 |
| Vedolizumb versus Ustekinumab | 1.154 | 0.337-3.946 | 0.820 | - | - | - |
| CD, Crohn's disease; TNF, tumour necrosis factor; CDAI, Crohn’s disease activity index; SES-CD, Simple endoscopic score for Crohn’s disease; HR, hazard ratio; CI, confidence interval  *Adjusted for age, sex, Montreal classification, Crohn’s disease activity index, and use of concomitant medications | | | | | | |

| **Supplementary Table 3. Predictors of clinical relapse in UC patients at 48 weeks (remission vs. response/non-response)** | | | | | | |
| --- | --- | --- | --- | --- | --- | --- |
|  | Univariate | | | Adjusted Response/Non-response | | |
| Variables |  |  |  | Multivariate* | | |
|  | HR | 95% CI | *P* value | HR | 95% CI | *P* value |
| Demographic variables |  |  |  |  |  |  |
| Age ≥ 40 years |  | ( Reference ) |  |  | ( Reference ) |  |
| Age < 40 years | 1.958 | 0.652-5.878 | 0.231 | 5.330 | 1.118-25.415 | 0.036 |
| Male gender | 1.467 | 0.450-4.778 | 0.525 | 2.388 | 0.623-9.159 | 0.204 |
| Body mass index | 0.895 | 0.725-1.105 | 0.301 | - | - | - |
| Duration from UC diagnosis, years | 0.989 | 0.949-1.032 | 0.619 | - | - | - |
| Smoking status at diagnosis |  |  |  |  |  |  |
| Never smoked |  | ( Reference ) |  |  | ( Reference ) |  |
| Ex-smoker & Current smoker | 1.094 | 0.354-3.376 | 0.876 | 2.125 | 0.530-8.523 | 0.287 |
| Previous intestinal resection surgery | 0.045 | 0.000-2578.056 | 0.580 | - | - | - |
| UC disease location |  |  |  |  |  |  |
| Proctitis (E1), Left sided (E2) |  | ( Reference ) |  |  | ( Reference ) |  |
| Pancolitis (E3) | 2.181 | 0.596-7.978 | 0.239 | 4.896 | 1.074-22.326 | 0.040 |
| Prior anti TNF therapy use |  |  |  |  |  |  |
| 1 |  | ( Reference ) |  |  |  |  |
| ≥2 | 0.322 | 0.070-1.470 | 0.143 | - | - | - |
| Disease activity index |  |  |  |  |  |  |
| Mayo score 6-10 |  | ( Reference ) |  |  |  |  |
| Mayo score 11-12 | 1.102 | 0.369-3.289 | 0.862 | - | - | - |
| Laboratory variables |  |  |  |  |  |  |
| Hemoglobin, g/dL | 0.909 | 0.567-1.458 | 0.692 | - | - | - |
| Erythrocyte sedimentation rate, mm/hr | 1.005 | 0.991-1.018 | 0.516 | - | - | - |
| Serum C-reactive protein, mg/dL | 1.000 | 0.982-1.019 | 0.976 | - | - | - |
| Serum albumin, g/dL | 0.683 | 0.287-1.630 | 0.391 | 0.434 | 0.112-1.677 | 0.226 |
| Concomitant medication |  |  |  |  |  |  |
| Steroid | 2.248 | 0.750-6.735 | 0.148 | 3.846 | 1.044-14.165 | 0.043 |
| Immunomodulator | 0.732 | 0.293-1.825 | 0.503 | - | - | - |
| Vedolizumab versus Tofacitinib | 1.693 | 0.494-5.803 | 0.402 | - | - | - |
| UC, ulcerative colitis; TNF, tumour necrosis factor; HR, hazard ratio; CI, confidence interval *Adjusted for age, sex, smoking status, disease location, serum albumin levels, and concomitant use of steroids | | | | | | |

| **Supplementary Table 4. Predictors of clinical relapse in CD patients at 48 weeks (remission vs. response/non-response)** | | | | | | |
| --- | --- | --- | --- | --- | --- | --- |
|  | Univariate | | | Adjusted Response/Non-response | | |
| Variables |  |  |  | Multivariate* | | |
|  | HR | 95% CI | *P* value | HR | 95% CI | *P* value |
| Demographic variables |  |  |  |  |  |  |
| Age ≥ 40 years |  | ( Reference ) |  |  | ( Reference ) |  |
| Age < 40 years | 1.472 | 0.642-3.374 | 0.361 | - | - | - |
| Male gender | 0.607 | 0.268-1.372 | 0.230 | - | - | - |
| Body mass index | 0.897 | 0.758-1.061 | 0.204 | 0.872 | 0.694-1.092 | 0.233 |
| Duration from diagnosis, year | 0.990 | 0.949-1.033 | 0.651 | - | - | - |
| Smoking status at diagnosis |  |  |  |  |  |  |
| Never smoked |  | ( Reference ) |  |  | ( Reference ) |  |
| Ex-smoker | 0.502 | 0.116-2.174 | 0.356 | - | - | - |
| Current smoker | 1.053 | 0.244-4.549 | 0.945 | - | - | - |
| Previous intestinal resection surgery | 1.011 | 0.400-2.553 | 0.982 | 0.422 | 0.098-1.821 | 0.247 |
| Montreal location |  |  |  |  |  |  |
| Ileal (L1), Colonic (L2), Isolated upper GI disease (L4) | | ( Reference ) |  |  |  |  |
| Ileocolonic (L3) | 1.597 | 0.593-4.297 | 0.354 | - | - | - |
| Montreal disease behavior |  |  |  |  |  |  |
| Nonstricturing, nonpenetrating (B1) |  | ( Reference ) |  |  |  |  |
| Stricturing (B2), Penetrating (B3) | 1.101 | 0.432-2.806 | 0.841 | - | - | - |
| Perianal disease modifier (p) | 2.130 | 0.727-6.240 | 0.168 | - | - | - |
| Prior anti TNF therapy use |  |  |  |  |  |  |
| 1 |  | ( Reference ) |  |  |  |  |
| ≥2 | 1.229 | 0.548-2.756 | 0.617 | - | - | - |
| Crohn's disease activity index |  |  |  |  |  |  |
| 220 ≤ CDAI < 450 |  | ( Reference ) |  |  |  |  |
| CDAI ≥ 450 | 0.322 | 0.043-2.402 | 0.269 | - | - | - |
| Endoscopic disease activity |  |  |  |  |  |  |
| SES-CD score 3-6 |  | ( Reference ) |  |  |  |  |
| SES-CD score 7-15 | 0.723 | 0.303-1.728 | 0.466 | - | - | - |
| SES-CD score > 15 | 1.739 | 0.354-8.545 | 0.496 | - | - | - |
| Laboratory variables |  |  |  |  |  |  |
| Hemoglobin, g/dL | 1.222 | 0.851-1.753 | 0.277 | 1.516 | 0.953-2.412 | 0.079 |
| Erythrocyte sedimentation rate, mm/hr | 1.001 | 0.992-1.009 | 0.899 | - | - | - |
| Serum C-reactive protein, mg/dL | 1.018 | 1.005-1.032 | 0.006 | 1.036 | 1.016-1.055 | <0.001 |
| Serum albumin, g/dL | 1.081 | 0.647-1.807 | 0.767 | 0.680 | 0.265-1.740 | 0.421 |
| Concomitant medication |  |  |  |  |  |  |
| Steroid | 3.783 | 1.292-11.080 | 0.015 | 8.448 | 2.155-33.113 | 0.002 |
| Immunomodulator | 0.865 | 0.577-1.295 | 0.480 | - | - | - |
| Vedolizumb versus Ustekinumab | 0.828 | 0.325-2.108 | 0.692 | 2.020 | 0.677-6.024 | 0.207 |
| CD, Crohn's disease; TNF, tumour necrosis factor; CDAI, Crohn’s disease activity index; SES-CD, Simple endoscopic score for Crohn’s disease; HR, hazard ratio; CI, confidence interval  *Adjusted for body mass index, previous intestinal resection, laboratory variables, concomitant use of steroids, and use of vedolizumab versus ustekinumab | | | | | | |
